# Measuring Dilution of Microbicide Gels with Optical Imaging

**DOI:** 10.1371/journal.pone.0082213

**Published:** 2013-12-10

**Authors:** Tyler K. Drake, Tejen Shah, Jennifer J. Peters, Adam Wax, David F. Katz

**Affiliations:** 1 Duke University, Department of Biomedical Engineering, Durham, North Carolina, United States of America; 2 Duke University, Department of Obstetrics and Gynecology, Durham, North Carolina, United States of America; University of Illinois at Chicago, United States of America

## Abstract

We present a novel approach for measuring topical microbicide gel dilution using optical imaging. The approach compares gel thickness measurements from fluorimetry and multiplexed low coherence interferometry in order to calculate dilution of a gel. As a microbicide gel becomes diluted at fixed thickness, its mLCI thickness measurement remains constant, while the fluorimetry signal decreases in intensity. The difference between the two measurements is related to the extent of gel dilution. These two optical modalities are implemented in a single endoscopic instrument that enables simultaneous data collection. A preliminary validation study was performed with *in vitro* placebo gel measurements taken in a controlled test socket. It was found that change in slope of the regression line between fluorimetry and mLCI based measurements indicates dilution. A dilution calibration curve was then generated by repeating the test socket measurements with serial dilutions of placebo gel with vaginal fluid simulant. This methodology can provide valuable dilution information on candidate microbicide products, which could substantially enhance our understanding of their *in vivo* functioning.

## Introduction

Microbicide gels are topically acting products that are inserted into the vagina to inhibit infection by sexually transmitted pathogens, mainly HIV. There have been mixed results in the successes of multiple Phase 3 microbicide trials of such gels [1], [2]. These trials evaluated gels that were not designed using knowledge of their intravaginal spreading mechanics and its effects upon drug delivery and retardation of viral migration to epithelial surfaces. Such information can contribute to the rational design of improved microbicide gel products. This knowledge is derived from a combination of experimental measurements relating to vaginal deployment and drug delivery [3], [4], and objective computations that relate product properties and dosage regimens to drug delivery [5]–[8]. These measurements include rheological properties of gels, drug release rates, and also the effects of contact with and dilution by ambient vaginal fluids.

Dilution of gel formulations changes their rheological properties and also the transport properties (e.g. diffusion coefficients) of both their microbicide drugs and virions migrating through them [5]–[7]. Thus, dilution may have a substantial effect upon the functioning of vaginal microbicide gels. However, the actual extent and rate of gel dilution within the human vagina *in vivo* are not known. The amount of vaginal fluid is believed to vary with a number of factors, primarily related to a women's reproductive endocrine status, that is the phase of the menstrual cycle and age. However, it has been very difficult to measure the actual amount of ambient fluid within the human vagina. This amount has been estimated to be 0.5–0.75 mL [9]. As a result, for example, a gel volume ranging from 2–5 mL would be potentially diluted by about 10–30% [5]. Presently, there is no methodology that can directly measure the amount and distribution of vaginal fluid, or gel dilution, *in vivo*. Clearly, there is a great need to develop and apply such methodology. Here, we present an optical technique that offers promise in performing measurements of intravaginal gel dilution in women *in vivo*.

Previous studies have successfully measured *in vivo* vaginal gel thickness distributions in a human clinical study using a probe-based, dual modality optical imaging instrument [3], [4], [10], [11]. Here, we exploit differences in the instrument's two optical imaging modalities in order to measure dilution of a placebo microbicide gel. The optical imaging instrument uses both multiplexed low coherence interferometry (mLCI) and fluorimetry in order to measure local gel coating thickness [10]. The mLCI method exploits low coherence light to perform depth-ranging measurements of layers in a sample (analogous to ultrasound), while fluorimetry requires the gel to be labeled with sodium fluorescein and measures the intensity of emitted light from the fluorophore. Therefore, as a gel becomes diluted at fixed thickness, its mLCI measurement changes minimally while its fluorimetry signal becomes less intense. The difference between these two types of measurements can thus be related, in principle, to the extent of dilution. This approach would provide a method of quantifying the amount of dilution of a microbicide gel product within the vaginal canal.

In the initial study here, we demonstrate the feasibility of the technique by conducting serial dilution experiments and comparing measurements between the two optical imaging modalities. It is hypothesized that mLCI measurements will vary slightly with dilution, as mLCI thickness measurements rely on index of refraction of the material (gel) which will be altered by dilution. This hypothesis is tested in this manuscript and the significance of changes in index of refraction is quantified and discussed. The methodology is then applied to sample *in vivo* gel thickness data as an example of how the approach may be used in understanding microbicide gel properties.

## Methods

### Optical Imaging Instrument

Complete descriptions of the dual-modality optical imaging instrument are provided in our previous papers [3], [4], [10], [11]. Below, we present the important operational features as used in this study.

The optical sensors of the dual-modality instrument are contained within an optically clear, 27-mm diameter, molded epoxy tube (Epoxy Technology, Epo-Tek 301, Billerica, MA). The tube is 150-mm long, with a wall thickness of 3.1mm, and allows the instrument's imaging optics to freely rotate and translate inside. The fluorimetry sensor is built around a 4-mm diameter endoscope (Storz 27005CA, Tuttlingen, Germany) that both delivers fluorescence excitation light to the sample and collects the emitted signal. The fluorimetric probe tip is built into a sealed well filled with distilled water to reduce spurious reflections. Fluorescence excitation is provided by a 300-watt Xenon lamp which is notch-filtered to remove the emission wavelength of fluorescence, at 500 to 560 nm). In this experiment, the collected fluorescence was split into two signals with a beam splitter. One was passed through a fiber optic cable and its intensity was detected using a photomultiplier tube. The second signal was imaged with a video camera to provide visual reference of probe position.

**Figure 1:**
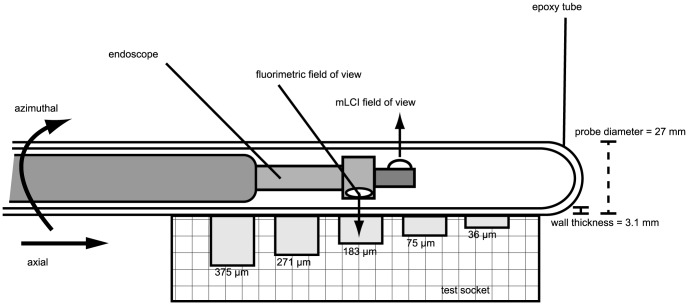
Imaging probe geometry as used with the test socket. The internal endoscope, which includes both the mLCI and fluorimetry imaging systems, rotates together freely inside the epoxy tube. The mLCI field of view is offset +20-mm in the axial direction and 180-degrees azimuthally. The test socket consists of grooves at five depths, ranging from 36–375 *µ*m.

The mLCI imaging module is mounted to the distal end of the fluorimetric endoscope such that the imaging area is offset 180-degrees and 20-mm axially, as shown in Fig. 1. This arrangement was necessary as the mLCI was added to the fluorimetry probe after its initial design [10]. Future studies will employ a redesigned instrument that does not include the axial offset. The mLCI instrument is constructed of 6-parallel interferometer channels which each provide depth-resolved reflectance profiles of a sample, analogous to acoustic reflection profiles of ultrasound. The mLCI instrument uses broadband light from a superluminescent diode (SLD, Superlum Diodes Ltd., Moscow, Russia) with a center wavelength of 837.5 nm and a bandwidth of 54.2 nm, yielding a theoretical coherence length of 5.7 µm. The measured average axial resolution of the mLCI instrument is 8.3 µm [10]. SLD light is split into 6 channels by 50∶50 fiber optic couplers (AC Photonics, Santa Clara, CA), creating 6 parallel sample and reference fields. In each channel, the collected light from the sample is recombined with reference light by fiber optic couplers. The signals are then detected simultaneously with a multichannel spectrometer (Avantes, Inc., Broomfield, CO) at 48 scans/sec. Axial probe position inside the epoxy tube is provided by a linear encoder system while a rotational encoder provides azimuthal position. These data are then used to transform register the data between the two modalities.

### Experimental Design

We created a custom cylindrical calibration socket for use in simultaneously evaluating mLCI and fluorometric measurements in relation to gel dilution and thickness. The test socket is made of black anodized aluminum and features 5 grooves of varying depths (36, 75, 183, 271, and 375 µm). These depths are consistent with *in vivo* gel thicknesses that have been detected in previous studies [3], [4]. The test gel was the placebo used in the current FACTS-001 trial [12]. This is a 3% hydroxyethyl cellulose (HEC) gel. The gel was serially diluted using vaginal fluid simulant [9] by amounts (v∶v) of 0% (whole gel) to 10%, 20%, 33%, and 50%.

An initial experiment was performed with unlabeled gel in order to: (1) determine the index of refraction of the gel at each dilution, and (2) characterize light leakage of the fluorimetry measurement system. The five grooves of the test socket were first filled with undiluted gel. The socket was secured to the epoxy tube of the probe, and imaged with the dual-modality instrument. The mLCI dataset was used to calculate index of refraction, *n*, of each dilution. The measured optical path length (*OPL*) of a mLCI depth scan (A-scan), can be related to index of refraction by OPL = n*t where *t* is the depth of a groove in the test socket. Fluorimetric light leakage leads to an overestimate of actual signal and was characterized in this preliminary experiment as well. The fluorimetric signal was recorded for each groove during the scan process of the unlabeled gel, and this intensity value was subtracted from the fluorescein-labeled gel signal in the remaining experiments.

Fluorescein-labeled test gel (0.1% w/w; Akorn AK-Fluor, Lake Forest, IL) was then inserted into the test socket and the five wells were imaged. The labeled gel was then diluted 10% by volume with VFS, placed into the socket, and imaged. This procedure was repeated for dilutions of 20%, 33%, and 50% VFS, with simultaneous measurements by fluorimetry and mLCI for each.

Fluorimetry measurements were taken at the center coordinate of each groove in the socket, with a field of view of approximately 1 cm. The mLCI field consisted of six 50 µm-diameter spots oriented in a line perpendicular to the socket's axis, covering about 3.5 mm azimuthally. mLCI data were sampled every 1-mm axially, and 10 axial measurements were averaged in each depth groove of the test socket for data comparison between the two modalities.

### Pilot Application to Human In Vivo Imaging Data

Application of the differential analysis of local mLCI and fluorimetry measurements to human *in vivo* data has many details that are beyond the scope of this initial, proof of principle study. For example, the noise inherent in the human data needs to be filtered in a manner that does not obfuscate distinctions in data from the two measurement modalities. Nonetheless, to demonstrate the potential of this approach we conducted a pilot analysis using a dataset from our previous *in vivo* study [3]. This was obtained 10 minutes after insertion of 4 mL of the same placebo gel used in the *in vitro* experiments described above. The dataset contains a two dimensional surface map of *in vivo* vaginal measurements of the thickness of gel in cylindrical coordinates (distance along the axis of the probe, and azimuthal angle). To measure an effective gel dilution, the local measurements were first azimuthally averaged, in relation to axial distance from the vaginal fornix to the introitus, as in Drake *et al* [3]. Then a region of interest (ROI) was selected, based on constraints of the mLCI method for very large gel coating thicknesses, which occur for a small region of the gel within the inner vaginal fornix [3], [13]. mLCI and fluorimetry data within the ROI were then plotted and regression analysis gave the slope, which was then used to infer a spatial average gel dilution within the ROI.

## Results

### Analysis of the In Vitro Experiments

Optical path length measurements obtained by mLCI were first used to calculate index of refraction, *n*, for each gel dilution with the equation OPL = n*t, where *t* is the known thickness of the calibration socket. These results are presented in Table 1, and were used to calibrate the mLCI device for consequent measurements with fluorescein-labeled FACTS001 gel thickness measurements.

**Table 1:** Calculated index of refraction values (*n*) for FACTS-001 gel diluted with vaginal fluid simulant. These values were used for accurate calibration of the mLCI modality.

Gel Dilution (% VFS)	0	10	20	33	50
***n***	1.43	1.41	1.40	1.40	1.37

**Figure 2:**
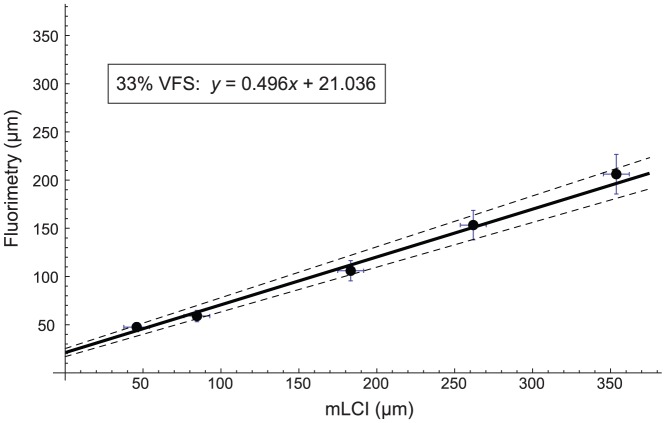
Gel thickness measurements of 33% VFS dilution of FACTS-001 gel with vaginal fluid simulant in the calibration socket as measured by fluorimetry (y-axis) and mLCI (x-axis). A refractive index value of 1.4 (Table 1) was used to calculate mLCI thickness. Line of best fit was found using a weighted least-squares algorithm. The 95% confidence interval for the fitted line is provided by the dashed lines.

Thickness measurements of the 33% VFS diluted, fluorescein-labeled test gel in the test socket are plotted below in Fig. 2, as an example. The mLCI error (x-axis) is given by the average axial resolution of the modality, 8.3 µm [10]. Fluorimetric error is defined as ±10% as described by Henderson *et al* [4]. Since varying degrees of error exist, as well as error in *both* coordinates, the traditional linear least squares method of regression is not applicable. For example, if the x-coordinates were measured with no error, the line of best fit would be the line that minimizes the sum of squares of the y-coordinate residuals. Instead, a weighted orthogonal least-squares regression was performed, based on Krysteck and Anton [14]. This minimizes residuals in both coordinates and accounts for the varying degree of error in fluorimetry measurements. An algorithm similar to Kysteck and Anton was developed in Mathematica (Wolfram Research, Inc.) which minimizes the equation,
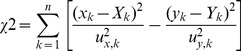
(1)where (*x_k_, y_k_*) are the *n* data pairs with uncertainties (*u_x,k_, u_y,k_*), and (*X_k_, Y_k_*) are the points of a straight line given by *y = mx+b* [14]–[16]. The algorithm was first verified by using the example data set provided by York [16]. The line of best fit for 33% VFS dilution is plotted below in Fig. 2. The dashed lines represent the confidence interval of the fit and are given by the equation,
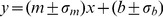
(2)where σ_m_ and σ*_b_* are the standard deviation in the slope and intercept, respectively.

**Figure 3:**
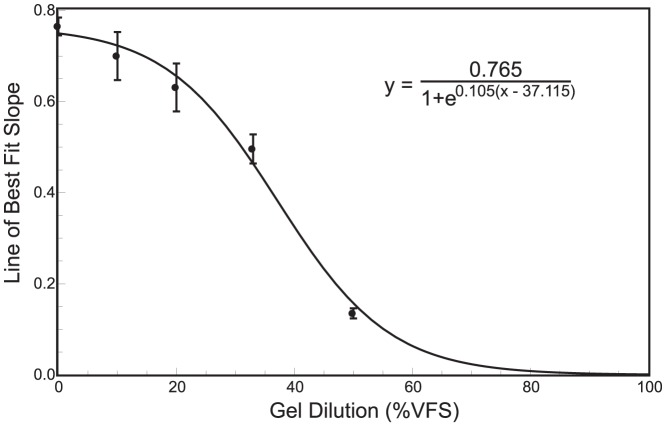
Line of best fit slope (from weighted total linear regression in Fig. 2) plotted against gel dilution (% VFS). The sigmoid fit was determined with a logistic 4-parameter analysis, and the resultant equation is displayed in the figure.

Regression slope values were calculated for each dilution (Table 2) and plotted against percentage dilution by volume of gel with VFS in Fig. 3. The data were fit with a sigmoid curve by using a logistic four parameter fit. The maximum asymptote was given by the slope of 0% VFS dilution and the minimum asymptote was set to zero. The inflection point and slope factor were then determined by an iterative optimization process within the statistical software, JMP Pro (SAS Institute, Cary, NC). The slope factor was determined to be 0.105 (S.E. = 0.012) and the inflection point was found to have a value of 37.115 (S.E. = 1.24). The y-axis error bars represent the standard deviation of slope measurements shown in Table 2.

**Table 2:** Results from weighted least-squares regressions performed on each dilution (SD =  standard deviation).

Gel Dilution (% VFS)	Line of best fit	SD of slope	SD of intercept
**0**	y = 0.765x+15.848	0.019	2.998
**10**	y = 0.700x+27.889	0.056	7.465
**20**	y = 0.631x+36.149	0.053	7.761
**33**	y = 0.496x+21.036	0.032	4.190
**50**	y = 0.135x+33.562	0.011	1.755

Strictly speaking, dilution at each gel thickness depends upon both the slope of the regression line and the intercept. We investigated an algorithm for deducing dilution in which the effects of the intercept, *b*, on prediction of dilution are neglected. As such, a universal relationship can be created that predicts dilution on the basis of the slope of the regression line. In order to calculate dilution values from a given slope value, the equation from Fig. 3 was inverted. Solving for *x* yields:
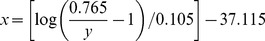
(3)


An error analysis of Equation 3 was performed to assess its fidelity in predicting dilution. Results are provided in Table 3. First, slope values, *m*, which resulted from the weighted least squares linear fit (Table 2) were used to inversely calculate % VFS dilution. A similar analysis was also performed with *m*±*SD* to test the response and sensitivity of Equation 3.

**Table 3:** Error Analysis of dilution calculation derived from the slope of the line of best fit in Figure 3. SD =  standard deviation.

Gel Dilution (% VFS)	Slope of Line of best fit (*m*±*SD*)	Calculated Dilution(% VFS)	Slope - SD Calculated Dilution (%VFS)	Slope + SD Calculated Dilution (%VFS)	
**0**	0.765±0.019	0	0	2.4	
**10**	0.700±0.056	14.5	5.4	21.2	
**20**	0.631±0.053	22.4	16.8	26.3	
**33**	0.496±0.032	31.3	29.5	33.0	
**50**	0.135±0.011	51.8	50.9	52.8	

### Pilot Application to Human In Vivo Imaging Data

**Figure 4:**
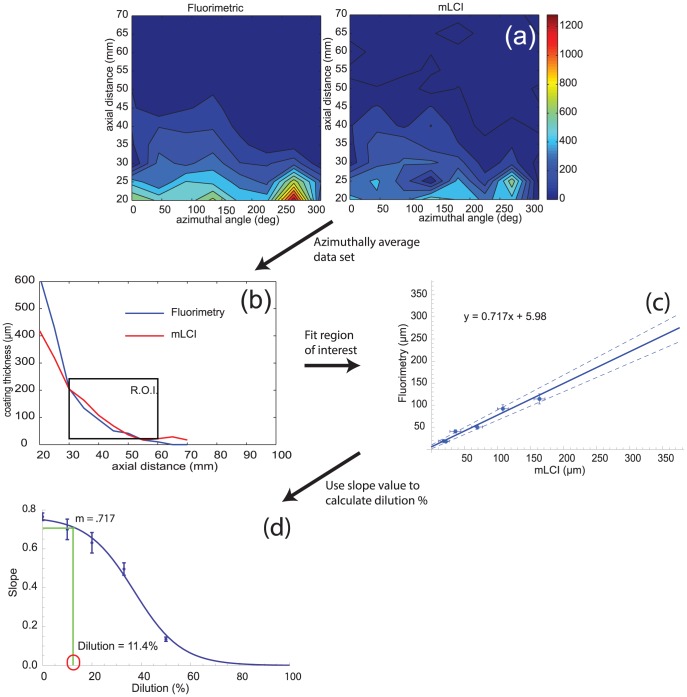
Example *in vivo* microbicide gel dilution calculation to demonstrate the complete method. Part (a) displays a topological plot of data obtained by the dual-modality optical imaging instrument. These data are azimuthally averaged as shown in (b) and a region of interest is selected. The dataset is then plotted in (c) and a weighted least-squares fit is performed on the values in the ROI to find the slope value of the regression. This slope value is then used with the calibration curve (Fig. 3) to find a dilution value of 11.4±10.5%. The topological data in (a) and (b) were originally presented in Drake *et al.* [3], [13]

Figure 4(a) shows the surface map of the local coating thickness distributions as measured *in vivo* by fluorimetry and mLCI for gel deployed in the vagina. Figure 4(b) shows the azimuthally averaged data plotted versus position along the axis of the probe (and thus, along the axis of the vaginal canal). The region of interest (ROI) was selected visually for this pilot application of the method. Figure 4(c) shows the plot of the mLCI and fluorimetry data, which were fit to a line using the weighted least-squares method (Equation 1). The slope of this regression line was *m* = 0.717±0.072. This value was then used with Equation 3 to deduce a gel dilution value in the ROI of 11.4±10.5%.

## Discussion

After insertion into the vagina, microbicide gels contact *in vivo* fluids and may become progressively diluted over time. Dilution changes rheological and transport properties of the gel for both its active pharmaceutical ingredients and HIV virions that contact it. This in turn can alter details of gel function as a drug delivery vehicle and barrier to HIV. Dilution also affects the net vaginal surface area coated by a gel, and reduced gel viscosity due to dilution could exacerbate gel leakage [5], [6]. At present there is great concern that gel leakage or “messiness” is a significant impediment to user acceptance, which reduces adherence to designated gel application protocols in clinical trials [17]. Clearly, it is critical to characterize the extent to which microbicide gels become diluted *in vivo*, as part of characterizing their performances as potential HIV prophylactics.

In this study, we have presented an optical methodology for deducing the amount of dilution of a microbicide placebo gel. The HEC-based structure of this gel is very similar to that of the Tenofovir gel now in its third anti-HIV efficacy trial, FACTS-001. Analysis of our *in vitro* experiments, using serial dilutions of the gel with a vaginal fluid simulant, gave a positive result for use of the method in predicting gel dilution.

We also found that dilution changed the index of refraction of the gel, which affects the accuracy of mLCI measurements; however, this change was minimal. The calculated undiluted gel index of refraction was 1.43 and this dropped to 1.37 at 50% dilution with VFS, a change of 4%. We posit that effects of this change are not biologically significant, based upon what we currently know about the likelihood of gel dilution *in vivo*. Of course, this could be tested in future *in vivo* studies. Further, we note that the optical properties of multiple contemporary vaginal microbicide gels, while not strictly identical, would likely not preclude evaluation by this method using a simple calibration for each gel.

From Fig. 2, it can be seen that as gel layer thicknesses increases, the confidence interval of the linear fit also increases. This is due to the ±10% measurement error of the fluorimetry device, which reduces the accuracy of the linear fit at relatively thick gel layers. The sigmoid shape of the dilution curve in Fig. 3 reveals a non-linear response in the system. At low dilutions (<20%), the curve is comparatively flat, and perturbations in slope values result in proportionally larger changes in calculated dilution, as compared to the more linear response seen at the center section of the sigmoid curve. The same variation is true at large dilutions (>50%) where the dilution curve again flattens out as it approaches the minimum asymptote of zero.

Even with this acknowledged reduction in accuracy at very low and high gel dilutions, the instrument and methodology remain promising as an approach for measuring microbicide gel dilution, and assessing its consequences to gel function. For example, Lai *et al.* concluded that microbicide gel dilutions on the order of 10–30% by volume resulted in observable changes in behavior, such as rheological properties and coating flows [5]. The optical imaging instrument is capable of measuring dilution of a gel with fidelity that we believe is biologically relevant. From Table 3, the 10% VFS dilution had the largest standard deviation of the slope value and this worst-case scenario yielded a change in calculated dilution of 15.8%, within the margin of Lai *et al* [5]. Non-uniform dilutions of microbicide gels may well occur *in vivo*. Our dual-modality instrument is capable of measuring such dilution distributions since measurements are made every 5-mm axially and at 8 azimuthal angles while the probe is inserted and stationary, providing a local dilution mapping of the complete vaginal tissue surface. [3], [10].

A pilot methodology for computing the extent of microbicide gel dilution for human *in vivo* thickness distribution data was outlined in Fig. 4. An ROI was selected near the leading edge of the gel, 30–60 mm of axial distance, and a dilution of 11.4±10.5% was calculated. This is a spatially averaged dilution measurement, and is consistent with calculations by Lai *et al.* of expected dilution *in vivo*. We believe that the value of 11% is reasonable because it falls in the range provided by Lai *et al.*; but without a “gold standard” of dilution measurement at the same specific *in vivo* location, we cannot know definitively [5]. The example was provided to illustrate the methodology. Clearly, there is much to do in implementing and validating this methodology with *in vivo* data. For example, the sensitivity in deducing dilution will need to be reconciled with the noise seen in the human data. We have begun this exercise, using data from a new human imaging study that is using an improved version of the imaging device that was applied in our original human studies.

## Conclusions

In summary, we have developed a method for assessing the dilution of microbicide gels using an optical imaging platform that we had previously developed and validated for measuring *in vivo* microbicide gel thickness distributions [3], [4], [10], [11], [18]. It was found that as gels become dilute, fluorimetry signals from the gel decrease while the mLCI signals, direct measures of physical thickness, remain constant. By using a controlled series of gel dilutions *in vitro*, a calibration curve was constructed which enabled deductions of dilution based on the two optical measurements. The accuracy of the measurements was determined to be sufficient for biologically relevant analyses of the influence of dilution on rheological properties and coating flows of vaginal microbicide and other gels. Ongoing clinical studies of vaginal microbicidal gel coating will examine the presence of dilution using the methodology developed here.
